# Postponed Items

**Published:** 1850-04

**Authors:** 


### THE WESTERN JOURNAL.

THIRD SERIES.—VOL. V.—NO. IV.

LOUISVILLE, APRIL 1, 1850.

---

POSTPONED ITEMS.

We regret that several editorial articles have been crowded out of this number. Among them are notices of Professor Bigelow's lecture, and Dr. Dowler's recent researches on the subject of death.

